# Impacto da COVID-19 na mortalidade domiciliar no Município do Rio de Janeiro, Brasil: análise temporal e espacial, de 2010 a 2020

**DOI:** 10.1590/0102-311XPT017524

**Published:** 2025-05-23

**Authors:** Nathalie Rodrigues Pontes Azevedo, Reinaldo Souza-Santos, Rosa Maria Soares Madeira Domingues

**Affiliations:** 1 Escola Nacional de Saúde Pública Sergio Arouca, Fundação Oswaldo Cruz, Rio de Janeiro, Brasil.; 2 Instituto Nacional de Infectologia Evandro Chagas, Fundação Oswaldo Cruz, Rio de Janeiro, Brasil.

**Keywords:** Análise Espacial, Mortalidade, COVID-19, Spatial Analysis, Mortality, COVID-19, Análisis Espacial, Mortalidad, COVID-19

## Abstract

O objetivo deste estudo é analisar a distribuição temporal e espacial da mortalidade domiciliar no Município do Rio de Janeiro, Brasil, antes e durante o primeiro ano da pandemia da COVID-19. Trata-se de estudo ecológico, em residentes com 15 anos ou mais, desenvolvido em duas etapas: (i) estudo de série temporal para analisar a taxa de mortalidade segundo local de ocorrência; a taxa de mortalidade domiciliar (TMD) segundo causa básica, idade e sexo; e a proporção de óbitos domiciliares por raça/cor e escolaridade, no período 2010-2020; e (ii) análise espacial para observar a variação espaço-temporal dos óbitos domiciliares de 2010 a 2020 por Região Administrativa (RA). Foram utilizados dados do Sistema de Informações sobre Mortalidade, estimativas populacionais e o Índice de Progresso Social (IPS), todos não identificados e de acesso público. Entre 2019 e 2020, houve aumento de 25,5% dos óbitos domiciliares, principalmente por causas infecciosas e parasitárias, transtornos mentais e causas mal definidas; aumento da TMD em todas as faixas etárias, exceto dos 15-19 anos; e aumento da proporção de óbitos domiciliares em pessoas de raça/cor preta e naqueles com até três anos de estudo. Foram detectados dois *clusters*: no primário (2016-2020), a maior TMD foi observada em RA com elevado IPS, enquanto no secundário (2020), as maiores TMD ocorreram em RA com menor IPS. Foi observado excesso de mortalidade no primeiro ano da pandemia da COVID-19 no Município do Rio de Janeiro. A mudança do perfil de casos e causas dos óbitos domiciliares e o aumento da TMD em RA com menor IPS sugerem aumento da mortalidade domiciliar em populações mais vulneráveis socialmente durante a pandemia.

## Abstract (en)

This study aimed to analyze the temporal and spatial distribution of home mortality in the municipality of Rio de Janeiro, Brazil, before and during the first year of the COVID-19 pandemic. It is an ecological study of residents aged 15 or older, developed in two stages: (i) Time-series analysis on mortality rates according to place of occurrence; home mortality rates (HMR) according to underlying cause, age, and sex; and the proportion of home deaths by race/color and educational level from 2010 to 2020, and; (ii) Spatial analysis to observe the spatiotemporal variation of home deaths from 2010 to 2020 by Administrative Region (AR). Data from the Brazilian Mortality Information System, population estimates and the Social Progress Index (SPI) were used, all of which are publicly available and anonymized. Between 2019 and 2020, home deaths increased by 25.5%, mainly due to infectious and parasitic diseases, mental disorders, and ill-defined causes. HMR increased in all age groups except 15-19 years, with a higher proportion of home deaths among black individuals and in those with up to three years of education. Two clusters were detected: the primary (2016-2020) showed the highest HMR in ARs with high SPI, while the secondary (2020) had the highest HMR in ARs with lower SPI. Excess mortality was observed in the first year of the COVID-19 pandemic in municipality of Rio de Janeiro. Changes in the profile and causes of home deaths, along with the rise of HRM in AR with lower SPI, suggest an increase in home mortality among socially vulnerable populations during the pandemic.

## Resumen

El objetivo de este estudio es analizar la distribución temporal y espacial de la mortalidad en los hogares en el municipio de Río de Janeiro, Brasil, antes y durante el primer año de la pandemia de la COVID-19. Se trata de un estudio ecológico en el cual participaron residentes de quince años o más y se desarrolló en dos etapas: (i) estudio de series temporales para analizar la tasa de mortalidad según el lugar de ocurrencia; la tasa de mortalidad en el hogar (TMH) según causa básica, edad y sexo; y la proporción de muertes en el hogar por raza/color y nivel de estudios en el período 2010-2020; y (ii) análisis espacial para observar la variación espaciotemporal de las muertes en el hogar de 2010 a 2020 por Región Administrativa (RA). Se utilizaron datos del Sistema de Informaciones de Mortalidad, estimaciones de población y el Índice de Progreso Social (IPS), todos no identificados y de acceso público. Entre 2019 y 2020 hubo un aumento del 25,5% en las muertes en el hogar, principalmente debido a causas infecciosas y parasitarias, trastornos mentales y causas mal definidas; un aumento en la TMH en todos los grupos de edad, excepto de los 15-19 años; y un aumento en la proporción de muertes en el hogar en personas de raza/color negro y en aquellas con hasta tres años de estudio. Se detectaron dos clústeres: en el primario (2016-2020), la TMH más alta tuvo RA con IPS alto, mientras que, en el secundario (2020), la TMH más alta se produjo en RA con IPS más bajo. Se observó un exceso de mortalidad en el primer año de la pandemia de la COVID-19 en el municipio de Río de Janeiro. El cambio en el perfil de casos y causas de muertes en el hogar y el aumento de la TMH en RA con menor IPS apuntan a un aumento de la mortalidad en el hogar en poblaciones más vulnerables socialmente durante la pandemia.

## Introdução

Em março de 2020 foi declarada a pandemia da COVID-19, uma doença infecciosa causada pelo SARS-CoV-2. Até 7 de janeiro de 2024, foram registrados 774 milhões de casos e 7 milhões óbitos no mundo, sendo mais de 37 milhões de casos e 705 mil óbitos no Brasil ^1^. Um excesso de mortalidade durante a pandemia foi relatado em vários países e esteve determinado não apenas por casos de COVID-19 ^2^ ^,^ ^3^ ^,^ ^4^ ^,^ ^5^. A pandemia também afetou a utilização dos serviços de saúde, efeito esse relatado em diversas áreas, tais como na atenção à saúde reprodutiva, pediatria, oncologia, doenças crônicas e serviços de emergência ^6^ ^,^ ^7^ ^,^ ^8^ ^,^ ^9^.

No Brasil, um estudo sobre o excesso de mortalidade por todas as causas em seis capitais brasileiras com maior incidência de COVID-19 durante as primeiras semanas da pandemia observou excesso de mortalidade em cinco cidades, das quais se destaca o Rio de Janeiro ^10^. Estudo posterior, que avaliou o excesso de mortalidade por todas as causas especificamente nesse município, nos dois primeiros anos da pandemia, encontrou incremento de 26,8% dos óbitos, distribuído em ondas, que seguiam os aumentos de incidência de casos de COVID-19 ^11^.

Além do excesso de mortalidade total, alguns estudos também identificaram aumento da mortalidade domiciliar durante o período da pandemia ^5^ ^,^ ^12^ ^,^ ^13^. No Brasil, o Estado de Minas Gerais, por exemplo, em relação à comparação do primeiro semestre dos anos 2019 e 2020, identificou aumento de óbitos domiciliares por diversas causas, tais como pneumonia, insuficiência respiratória, síndrome respiratória aguda grave, sepse, infarto agudo do miocárdio, acidente vascular cerebral e outras causas cardiovasculares ^12^. No País de Gales, dos óbitos em excesso que ocorreram durante a pandemia da COVID-19, mais da metade (54%) foram em ambiente domiciliar ^13^.

Entretanto, apesar das evidências existentes relativas ao excesso de mortalidade durante a pandemia da COVID-19, seus efeitos no local de ocorrência do óbito e no padrão de mortalidade domiciliar foram pouco investigados. Este estudo tem por objetivo analisar a frequência, a distribuição espacial, o perfil sociodemográfico e as causas de óbitos domiciliares, antes e durante o primeiro ano da pandemia da COVID-19, com o fim de identificar mudanças no perfil de mortalidade domiciliar que possam orientar a formulação de políticas públicas em eventos de saúde pública futuros.

## Métodos

### Desenho e contexto do estudo

Foi realizado um estudo ecológico no Município do Rio de Janeiro, capital do Estado do Rio de Janeiro, localizado na Região Sudeste do Brasil. O Município do Rio de Janeiro apresentava população estimada de 6.747.815 habitantes no ano 2020, sendo dividido em 10 Áreas Programáticas de Saúde (AP), 32 Regiões Administrativas (RAs) e 163 bairros.

O estudo foi desenvolvido em duas etapas:

(1) Estudo ecológico, de série temporal, tendo o Município do Rio de Janeiro como unidade de análise, para analisar: (i) a variação anual da taxa de mortalidade segundo local de ocorrência (hospital, domicílio, outro estabelecimento de saúde, via pública, outros); (ii) a variação da taxa de mortalidade domiciliar (TMD), específica por causa e características demográficas (idade e sexo); e (iii) a variação da proporção de óbitos domiciliares segundo escolaridade e raça/cor, no período 2010-2020;

(2) Estudo ecológico, de análise espacial, tendo as 32 RAs como unidade de análise, para verificar a distribuição de óbitos domiciliares segundo RA de residência em 2019 e 2020, e a variação espaço-temporal por meio da detecção de *clusters* das altas taxas de mortalidade domiciliar de 2010 a 2020 por RA.

### Participantes

Foram analisados todos os óbitos em maiores de 15 anos, por todas as causas, em residentes no Município do Rio de Janeiro.

### Fonte de dados

Foram utilizados dados secundários, não identificados, de uso público das seguintes fontes acessadas em abril de 2022:

(1) Sistema de Informações sobre Mortalidade (SIM) do TabNet da Secretaria Municipal de Saúde: contém informações sobre todos os óbitos ocorridos na cidade. Disponível em: http://tabnet.rio.rj.gov.br/cgi-bin/dh?sim/definicoes/sim_apos2005.def.

(2) Instituto Pereira Passos: (i) população residente no Município do Rio de Janeiro estimada por bairro e RA, segundo sexo e grupos etários. Disponível em: https://data.rio/documents/população-residente-estimada-e-projetada-por-sexo-e-grupos-etários-do-brasil-estado-do-rj-e-município-do-rio-de-janeiro-entre-1980-1991-2000-2065/about; e (ii) Índice de Progresso Social (IPS) 2020. Disponível em: https://ips-rio-pcrj.hub.arcgis.com/pages/2020. Indicador elaborado pelo Instituto Pereira Passos que consta de três dimensões e 12 componentes compreendidos nos aspectos das “necessidades humanas básicas”, “fundamentos do bem-estar” e “oportunidades”. Apresenta variação de 0 a 100, sendo os valores mais elevados indicativos de melhor condição social ^14^. No Município do Rio de Janeiro, a variação do IPS foi de 42,06 a 85,04 em 2020.

#### Variáveis

Foram utilizadas as seguintes variáveis:

(1) Óbitos em maiores de 15 anos segundo sexo (masculino, feminino), faixa etária em anos (15-19, 20-29, 30-39, 40-49, 50-59, 60-69, 70-79, 80 ou mais), cor da pele (branca, parda, preta, amarela, indígena, não informada), escolaridade em anos de estudo (nenhuma, 1-3, 4-7, 8-11, 12 ou mais), causa (por capítulo da Classificação Internacional de Doenças - 10ª revisão [CID-10], com o objetivo de agrupar um número menor de categorias de análise e facilitar a visualização dos resultados) e local de ocorrência (hospital, outro estabelecimento de saúde, domicílio, via pública);

(2) Ano de ocorrência do óbito (2010 a 2020);

(3) RA de residência do caso de óbito;

(4) IPS, utilizado para caracterização social das RAs.

#### Análise dos dados

##### Estudo de série temporal

Foi estimada a taxa de mortalidade segundo local de ocorrência do óbito no período 2010-2020. Posteriormente, foram calculadas as TMDs de residentes no Município do Rio de Janeiro, segundo causa, sexo e faixa etária, e as proporções de óbitos domiciliares, segundo raça/cor e escolaridade, por ano, para o período 2010-2020.

Foi utilizada a análise de regressão *joinpoint* para estudar a tendência temporal de mortalidade segundo local de ocorrência do óbito, bem como as tendências da TMD segundo causa, idade, sexo e da proporção de óbitos segundo raça/cor e escolaridade. Para esta análise foi utilizado o programa estatístico Joinpoint (https://surveillance.cancer.gov/joinpoint/) ^15^. Para as variáveis local de ocorrência, sexo, faixa etária e capítulo da CID-10 da causa básica, foram utilizadas as taxas de mortalidade. Para as variáveis raça/cor e escolaridade, foram utilizadas as proporções de óbitos. A análise foi realizada com o uso de variáveis dependentes “taxa” e “proporção” e de variáveis independentes os anos de 2010 a 2019. A regressão de Poisson foi utilizada para analisar a tendência observada no período 2010-2019, anterior à pandemia da COVID-19, com o cálculo da variação percentual anual observada no período. Em caso de identificação de pontos de inflexão durante a série temporal, foi calculada a variação percentual anual para cada segmento identificado. Para o cálculo da variação percentual 2019-2020 das taxas de mortalidade, utilizou-se a fórmula: (Taxa 2020 - Taxa 2019) / Taxa 2019 * 100. Para as variáveis raça/cor e escolaridade, a variação percentual 2019-2020 das proporções observadas foi calculada utilizando a fórmula: (Proporção 2020 - Proporção 2019) / Proporção 2019 * 100. Para identificar diferenças significativas na variação anual 2019-2020 em relação ao período pré-pandêmico, foi comparada a variação percentual observada entre 2019-2020 e a variação anual percentual no segmento mais recente identificado pela regressão *joinpoint*. Foi considerada uma diferença significativa se a variação anual 2019-2020 não estivesse contida no intervalo de confiança observado no período imediatamente anterior. Essa comparação foi feita para cada um dos aspectos analisados (local de ocorrência, capítulo de causa do CID-10, idade, sexo, raça/cor e escolaridade).

#### Análise espacial

A análise espacial foi conduzida utilizando-se as 32 RAs como unidade de agregação. Inicialmente, foram calculadas as TMDs por 100 mil habitantes, por RA de residência do caso de óbito, para os anos de 2019 e 2020, e elaborados mapas temáticos, um para cada ano, utilizando o sistema de informação geográfica QGIS, versão 3.20 (https://qgis.org/en/site/). A distribuição proporcional das RAs segundo faixas da TMD foi calculada para os dois anos analisados.

Posteriormente, para a análise espaço-temporal, foi utilizada a técnica de varredura pelo SatScan, versão 10.0 (http:\\www.satscan.org), para detectar *clusters* mais prováveis de altas TMDs no período de 2010 a 2020, considerando as RAs do Município do Rio de Janeiro, utilizando o modelo de Poisson. O *cluster* mais provável, considerado o *cluster* primário, apresenta o maior valor do resultado do teste de *log* da razão de verossimilhança (LRV), sendo estatisticamente significante (p < 0,05). Já o *cluster* secundário tem o segundo maior valor de verossimilhança, com valor de p < 0,05. Os parâmetros utilizados no SatScan consideraram 50% da população sob risco, valor aproximado da população que depende exclusivamente do Sistema Único de Saúde (SUS) no Município do Rio de Janeiro, e o raio foi definido automaticamente pelo software.

#### Aspectos éticos

A pesquisa está em conformidade com a *Resolução nº 510/2016*, do Conselho Nacional de Saúde, por utilizar informações de acesso público sob domínio compartilhado e obteve dispensa pelo Comitê de Ética em Pesquisa da Escola Nacional de Saúde Pública Sergio Arouca da Fundação Oswaldo Cruz (ENSP/FIOCRUZ; parecer nº 17/2021).

## Resultados

### Análise de série temporal

No período 2010-2019, foram registrados 624.233 óbitos em residentes no Município do Rio de Janeiro com 15 anos ou mais, com uma variação percentual anual significativa do número total de óbitos de 1,3%. Em 2020, foram registrados 81.208 óbitos de residentes com 15 anos ou mais no Município do Rio de Janeiro, o que corresponde a um aumento de 21% em relação ao total de óbitos em 2019. A TMD variou de 147,5 por 100 mil habitantes em 2010 a 166,0 por 100 mil habitantes em 2019, sendo a taxa mais baixa (136,9 por 100 mil habitantes) observada no ano 2012. Em 2020, a TMD alcançou o valor de 207,48 por 100 mil habitantes, um aumento de 25,5% em relação ao ano 2019.

Durante todo o período, a maioria dos óbitos (73%) ocorreu em hospitais, sendo o domicílio o segundo local mais frequente de ocorrência de óbitos (13%) no período 2010-2018. Em 2019, o segundo local mais frequente dos óbitos foi “outros estabelecimentos de saúde”, com os óbitos domiciliares retornando à segunda posição em 2020. No período 2012-2019 foi observado aumento dos óbitos domiciliares; e, no período 2010-2019, dos óbitos em outros estabelecimentos de saúde, sendo a maior variação percentual observada no período 2010-2013. Os óbitos em via pública apresentaram redução no período 2010-2016, enquanto os óbitos hospitalares não tiveram alterações no período. Ao comparar 2020 com 2019, a maior variação percentual anual foi observada nos óbitos ocorridos em via pública (112%), seguido dos óbitos em domicílio (25,5%) (Tabela 1).

**Tabela 1:** Variação percentual anual da taxa de mortalidade segundo local de ocorrência em residentes com 15 anos ou mais e da taxa de mortalidade domiciliar (TMD), segundo causa do óbito. Município do Rio de Janeiro, Brasil, 2010-2019 e 2019-2020.

Variáveis	Segmento *	Período		APC	Intervalo de confiança		Prob > |t|	Δ% 2019-2020
		Início	Final		Limite inferior	Limite superior		
Taxa de mortalidade segundo local de ocorrência do óbito								
Hospital	1	2010	2019	-0,1	-0,8	0,7	0,872	22,3
Outro estabelecimento	1	2010	2013	28,3 **	16,8	40,8	0,001	
Outro estabelecimento	2	2013	2019	9,1 *	5,7	12,6	0,001	18,1
Domicílio	1	2010	2012	-2,0	-10,1	6,8	0,570	
Domicílio	2	2012	2019	3,0 **	1,8	4,2	0,001	25,5
Via pública	1	2010	2016	-17,9 **	-26,0	-8,9	0,005	
Via pública	2	2016	2019	4,7	-23,1	42,4	0,718	112,0
Outros	1	2010	2019	2,9 **	0,9	5,0	0,010	-25,0
TMD específica por causa do óbito (capítulo CID-10)								
DIP	1	2010	2019	-3,2 **	-6,2	-0,1	0,046	381,0
Transtornos mentais	1	2010	2016	-7,8	-16,0	1,3	0,077	
Transtornos mentais	2	2016	2019	16,4	-11,7	53,6	0,217	111,7
Causas mal definidas	1	2010	2019	-0,9	-3,1	1,3	0,362	170,0
Neoplasia	1	2010	2019	-3,1 **	-4,7	-1,4	0,003	45,0
Causas externas	1	2010	2019	-0,7	-2,5	1,3	0,447	33,9
SNC	1	2010	2015	0,5	-4,0	5,2	0,803	
SNC	2	2015	2019	8,4 **	1,6	15,7	0,024	30,9
Doenças endócrinas	1	2010	2016	-5,4 **	-8,8	-1,8	0,012	
Doenças endócrinas	2	2016	2019	19,7 **	7,3	33,5	0,008	20,6
Aparelho digestivo	1	2010	2013	20,9 **	3,4	41,3	0,026	
Aparelho digestivo	2	2013	2019	-4,2	-9,2	0,9	0,088	4,8
Aparelho circulatório	1	2010	2012	-6,1	-17,3	6,6	0,256	
Aparelho circulatório	2	2012	2019	4,8 **	3,0	6,6	0,001	-3,0
Aparelho respiratório	1	2010	2016	8,7 **	5,5	12,1	0,001	
Aparelho respiratório	2	2016	2019	-9,9 **	-17,6	-1,4	0,031	-12,0

APC: variação percentual anual (do inglês, *annual percent change*); CID-10: 10ª revisão da Classificação Internacional de Doenças; DIP: doenças infecciosas e parasitárias; SNC: sistema nervoso central.

* Períodos identificados pela análise de tendência temporal;

** p < 0,05.

Durante todo o período de análise, os óbitos domiciliares por doenças do aparelho circulatório foram os mais frequentes (49,6% de 2010 a 2019, 42% em 2020), seguidos das neoplasias (10,2% de 2010 a 2019 e 10% em 2020) e das causas mal definidas do óbito (10,4% de 2010 a 2019 e 17,7% em 2020). No período observado, a taxa de mortalidade por doenças do aparelho circulatório apresentou aumento significativo no período 2012-2019, seguido das doenças endócrinas no período 2016-2019 e das doenças do sistema nervoso central (SNC) no período 2015-2019, enquanto a taxa de mortalidade por neoplasias e doenças infecciosas e parasitárias (DIP) apresentou redução no período 2010-2019; e por doenças respiratórias no período 2016-2019. Doenças do aparelho digestivo apresentaram aumento no período 2010-2013, seguida de estabilidade, enquanto causas mal definidas, causas externas e mentais não apresentaram alteração significativa no período. No ano 2020, todas as taxas de mortalidade específicas, exceto as doenças do aparelho circulatório e respiratório, apresentaram aumento. Os maiores aumentos percentuais foram observados nas taxas de mortalidade por doenças infecciosas, causas mal definidas e doenças mentais (Tabela 1).

No período 2010-2019 foi observado aumento significativo da TMD específica para ambos os sexos, sendo maior para o sexo feminino. No período 2019-2020, a variação anual percentual foi maior do que no período anterior para ambos os sexos, sendo mais elevada para residentes do sexo masculino (Tabela 2).

**Tabela 2:** Variação percentual anual da taxa de mortalidade domiciliar segundo sexo e idade e da proporção de óbitos domiciliares segundo raça/cor e escolaridade. Município do Rio de Janeiro, Brasil, 2010-2019 e 2019-2020.

Variáveis	Segmento *	Período		APC	Intervalo de confiança		Prob > |t|	Δ% 2019-2020
		Início	Final		Limite inferior	Limite superior		
Sexo								
Feminino	1	2010	2019	1,9 **	0,9	2,9	0,002	23,0
Masculino	1	2010	2019	1,3 **	0,3	2,3	0,014	2,7
Faixa etária (anos)								
15-19	1	2010	2019	3,2	-1,6	8,4	0,166	-33,5
20-29	1	2010	2019	1,4	-1,2	4,0	0,254	41,0
30-39	1	2010	2019	-2,0 **	-4,0	-0,1	0,045	34,1
40-49	1	2010	2019	-2,5 **	-3,5	-1,6	< 0,001	31,2
50-59	1	2010	2019	-1,6 **	-2,7	-0,5	0,011	36,0
60-69	1	2010	2012	-5,3	-15,1	5,6	0,257	
60-69	2	2012	2019	1,7 **	0,2	3,2	0,032	19,4
70-79	1	2010	2012	-4,8	-12,1	3,1	0,173	
70-79	2	2012	2019	0,2	-0,8	1,3	0,617	17,2
80 ou mais	1	2010	2019	0,5	-0,6	1,6	0,326	22,6
Cor da pele ***								
Branca	1	2010	2019	-0,8 **	-1,1	-0,6	< 0,001	-1,40
Preta	1	2010	2019	2,0 **	0,8	3,1	0,004	5,73
Parda	1	2010	2017	2,1 **	1,4	2,8	0,001	-0,64
Parda	2	2017	2019	-1,5	-6,5	3,7	0,478	
Amarela	1	2010	2019	-4,1	-12,4	4,9	0,314	112,46
Não informada	1	2010	2019	-8,5 **	-13,0	-3,7	0,004	66,17
Escolaridade (anos) ***								
Nenhuma	1	2010	2019	-0,2	-1,8	1,4	0,776	11,3
1-3	1	2010	2019	0,7	-2,6	4,2	0,648	10,4
4-7	1	2010	2019	-2,5 **	-4,7	-0,1	0,040	-20,1
8-11	1	2010	2019	2,5 **	1,9	3,1	< 0,001	2,7
12 ou mais	1	2010	2012	-6,9	-18,0	5,7	0,206	6,6
12 ou mais	2	2012	2019	2,1 **	0,3	3,8	0,028	

APC: variação percentual anual (do inglês, *annual percent change*).

* Períodos identificados pela análise de tendência temporal;

** p < 0,05;

*** Para as variáveis “raça/cor” e “escolaridade” foram utilizadas proporções.

A análise específica das taxas de mortalidade segundo faixa etária mostra uma redução significativa da TMD em pessoas de 30-39 anos, 40-49 anos e 50-59 anos no período 2010-2019, e aumento na faixa etária de 60-69 anos no período 2012-2019, sem alterações significativas nas demais faixas etárias. Em comparação a 2019, houve aumento da TMD em todas as faixas etárias em 2020, exceto na faixa de 15-19 anos, na qual foi observada redução. A maior variação percentual foi observada nas faixas abaixo de 60 anos, principalmente de 20-29 anos (aumento de 41%) e de 50-59 anos (aumento de 36%) (Tabela 2).

A proporção de óbitos domiciliares em residentes de cor da pele preta apresentou aumento no período 2010-2019 e redução para brancos. Entre 2010 e 2017, os óbitos de residentes de cor parda sofreram aumento significativo, com estabilidade entre 2017 e 2019. Os óbitos com raça cor “não informada” apresentaram redução no período de 2010-2019. Na comparação 2019-2020, observou-se aumento da proporção de óbitos em pretos, acima da variação anual observada no período 2010-2019, enquanto a variação em brancos e pardos não foi estatisticamente diferente da observada no período anterior. Os registros de óbitos com cor da pele não informada aumentaram no período 2019-2020, bem como em residentes de cor amarela (Tabela 2). Não foi realizada análise temporal dos óbitos em indígenas no período 2010-2019, devido à ausência de óbitos em alguns períodos. A variação entre 2019 e 2020 foi de 457,70 (de 1 óbito para 7 óbitos).

No período 2010-2019, observou-se uma redução significativa da proporção de óbitos domiciliares em residentes com 4-7 anos de estudo e estabilidade em residentes com 1-3 anos de estudo ou nenhuma escolaridade. Por outro lado, a proporção de óbitos domiciliares em pessoas com 8-11 anos de estudo apresentou aumento significativo no período 2010-2019, enquanto aumento significativo em pessoas com 12 ou mais anos de estudo foi observado a partir de 2012 (Tabela 2). No período 2019-2020, foi observado aumento da variação percentual em todas as faixas de escolaridade, exceto na faixa de 4-7 anos, na qual foi verificada redução superior à observada nos anos anteriores. A maior variação percentual foi observada em pessoas sem escolaridade e com 1-3 anos de estudo (11,3% e 10,4%, respectivamente), seguido de um aumento na variação percentual de 2019 para 2020 na faixa de 12 anos ou mais de estudo (6,6%) e de um aumento não significativo na faixa de 8-11 anos de estudo (2,7%) (Tabela 2).

### Análise espacial

**Figura 1:**
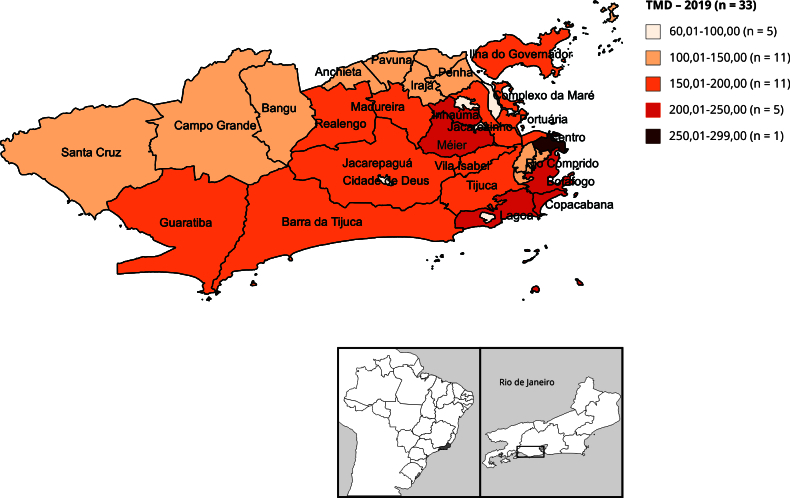
Taxa de mortalidade domiciliar (TMD) por 100 mil habitantes por Região Administrativa. Município do Rio de Janeiro, Brasil, 2019.

Foi verificada modificação da distribuição da mortalidade domiciliar nas RAs entre 2019 e 2020 (Figuras 1 e 2). Em 2019, 18,18% das RAs apresentavam TMD superior a 200 por 100 mil habitantes, enquanto em 2020 esse valor passou para 51,51%. Na varredura de altas taxas de mortalidade nas RAs (Figura 3), observamos a detecção de *clusters* em dois momentos. O *cluster* primário teve início no ano de 2016 e término em 2020, com LRV = 621,19. O raio deste *cluster* inclui as RAs Portuária, Centro, Rio Comprido, Botafogo, Copacabana, Lagoa, São Cristóvão, Tijuca, Vila Isabel, Ramos, Inhaúma, Méier, Santa Teresa e Jacarezinho. O *cluster* secundário, com LRV = 159,85, apresenta altas taxas pontualmente em 2020 e seu raio reúne as RAs Irajá, Madureira, Jacarepaguá, Bangu, Campo Grande, Anchieta, Barra da Tijuca, Pavuna, Guaratiba, Realengo e Cidade de Deus. No *cluster* primário, 2016-2020, a maior TMD (298,86 por 100 mil habitantes) foi observada na RA Copacabana, que possui o segundo maior IPS do Município do Rio de Janeiro (80,23). Já a segunda maior TMD (282,44 por 100 mil habitantes) foi detectada no *cluster* secundário, em 2020, em Guaratiba, RA com IPS de 43,54 (Tabela 3).

**Figura 2:**
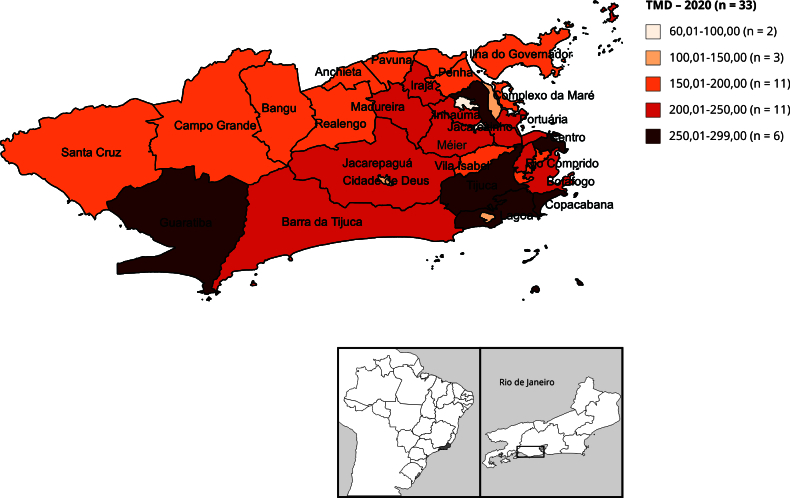
Taxa de mortalidade domiciliar (TMD) por 100 mil habitantes por Região Administrativa. Município do Rio de Janeiro, Brasil, 2020.

**Figura 3:**
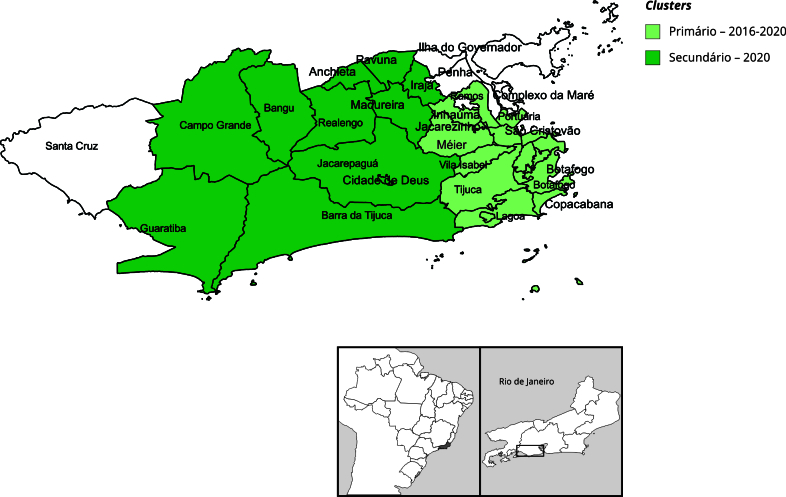
Detecção de *clusters* para altas taxas de mortalidade domiciliar (TMD) por Região Administrativa. Município do Rio de Janeiro, Brasil, 2010 a 2020.

**Tabela 3:** Taxa de mortalidade domiciliar (TMD) por 100 mil habitantes e Índice de Progresso Social (IPS), por Região Administrativa (RA), segundo a detecção dos *clusters*. Município do Rio de Janeiro, Brasil, 2020.

*Cluster*	RA	TMD	IPS (2020)
1	Portuária	216,77	42,06
1	Centro	269,10	55,59
1	Rio Comprido	214,14	55,50
1	Botafogo	242,36	85,03
1	Copacabana	298,86	80,23
1	Lagoa	268,96	79,02
1	São Cristóvão	208,63	51,86
1	Tijuca	256,51	71,61
1	Vila isabel	198,71	73,05
1	Ramos	276,28	57,04
1	Inhaúma	236,60	56,92
1	Méier	237,69	64,61
1	Santa Teresa	195,39	63,26
1	Jacarezinho	84,66	45,15
2	Irajá	231,14	65,57
2	Madureira	226,19	55,61
2	Jacarepaguá	212,14	61,94
2	Bangu	157,81	51,84
2	Campo Grande	186,86	58,68
2	Anchieta	181,31	55,72
2	Barra da Tijuca	206,78	69,70
2	Pavuna	172,66	42,97
2	Guaratiba	282,44	43,54
2	Realengo	177,51	55,76
2	Cidade de Deus	131,24	47,84

## Discussão

Verificou-se um aumento de 21% na mortalidade de residentes no Município do Rio de Janeiro maiores de 15 anos no ano de 2020, primeiro ano da pandemia da COVID-19. Esse aumento foi observado em todos os locais de ocorrência, sendo maior para os óbitos em via pública e no domicílio. Especificamente para os óbitos domiciliares, foi verificada mudança do perfil dos casos e das causas de óbito em relação ao período pré-pandêmico, com alteração da distribuição dos óbitos segundo faixa etária, sexo, raça/cor, escolaridade, causa básica e RA de residência.

Excesso de mortalidade durante os dois primeiros anos da pandemia da COVID-19 (2020-2021) foi observado em todo o mundo, com estimativa de que, globalmente, tenha sido 3,07 maior do que o registrado ^2^. Maior excesso de mortalidade foi observado em países em desenvolvimento e de média renda, quando comparados a países desenvolvidos e de alta renda ^3^. Diferenças em relação ao local do óbito também foram registradas em diferentes contextos. Em estudo realizado no Reino Unido ^5^, foi observado aumento de 41% na mortalidade domiciliar, principalmente em locais com menor privação ^16^, enquanto no México o excesso de mortalidade domiciliar foi de 145%, ocorrido principalmente em áreas de baixo nível socioeconômico ^17^. Neste estudo, o maior excesso de mortalidade foi observado em óbitos ocorridos em via pública (112%), seguido de óbitos em domicílio (25,5%).

De forma similar a outros estudos, maior excesso de mortalidade foi obervado em homens ^3^ ^,^ ^4^ ^,^ ^11^. Entretanto, foi identificado maior excesso de mortalidade domiciliar em indivíduos de 20 a 59 anos, enquanto em revisão sistemática incluindo estudos realizados em 79 países o maior excesso de mortalidade total foi observado na população com 60 anos ou mais ^3^. Em relação às causas dos óbitos domiciliares, o maior aumento foi observado para as causas infecciosas, causas mal definidas, transtornos mentais e óbitos por neoplasias. Esses resultados são coerentes com dados de outros países que também evidenciaram excesso de óbitos por todas as causas e não apenas pelas diretamente relacionadas à COVID-19 ^2^ ^,^ ^3^ ^,^ ^4^ ^,^ ^5^. O aumento de óbitos por causas mal definidas, que estavam estáveis no período pré-pandêmico, embora representassem a terceira causa de óbito domiciliar, sugere uma maior ocorrência de óbitos sem assistência médica. Aumento de óbitos por causas mal definidas também foi identificado em estudo de âmbito nacional no Brasil em 2020 ^18^. Já a maior mortalidade domiciliar por neoplasias, que estava em declínio no período 2010-2019, pode ser decorrente de dificuldades de acesso a serviços de saúde, mas pode também refletir a opção de manter um paciente terminal no domicílio, dadas as medidas restritivas no acesso de familiares adotadas pelos serviços hospitalares durante a pandemia. Em estudo realizado nos Estados Unidos ^8^, foi observada redução da utilização dos serviços de saúde por pacientes oncológicos, desde a realização de exames para diagnóstico e acompanhamento até internações hospitalares. O aumento dos óbitos por transtornos mentais pode ser explicado pelo estado de solidão, depressão, ansiedade e outros transtornos vivenciados durante o período da pandemia ^19^.

Uma hipótese para o aumento do número de óbitos no domicílio, bem como em vias públicas e em outros estabelecimentos de saúde observado no Município do Rio de Janeiro, seria a superlotação dos serviços hospitalares durante a pandemia da COVID-19. A fragilidade do sistema de saúde de países de baixa e média renda foi um fator que contribuiu para a ocorrência de mortalidade excessiva durante a pandemia da COVID-19, devido à demanda crescente por cuidados intensivos num contexto de recursos limitados, como respiradores, leitos de UTI (unidade de terapia intensiva) e medicamentos. Em contrapartida, países com sistemas de saúde mais robustos conseguiram de forma mais eficaz mitigar os impactos mais severos da pandemia, apesar de também terem enfrentado picos de mortalidade ^2^. No Brasil, Santos et al. ^20^, numa revisão integrativa que analisou 42 estudos sobre a oferta de leitos hospitalares no período de março a dezembro de 2020, identificaram desigualdades na distribuição regional da oferta de recursos e nos arranjos público-privados, inclusive em regiões com boa alocação de recursos hospitalares, impondo limitações para o enfrentamento da COVID-19 e o aprofundamento das desigualdades sociais no país. Essas desigualdades incluem o tempo de deslocamento para o acesso a leitos de UTI e a disponibilidade de transportes adequados, como UTI móvel.

Na análise espacial realizada neste estudo, foram identificados *cluster* primário de óbito domiciliar com início no ano de 2016 e *cluster* secundário, detectado pontualmente no ano de 2020. Ainda que este estudo seja ecológico, e que mudanças detectadas no nível agregado não possam ser atribuídas ao indivíduo, uma hipótese para esse resultado seria a mudança do perfil dos óbitos domiciliares observado nos dois períodos. O *cluster* primário foi identificado em RAs do Município do Rio de Janeiro que apresentam IPS mais elevado e espera-se que melhores condições socioeconômicas sejam facilitadoras do acesso aos serviços de saúde e cuidados no domicílio, seja por planos de saúde ou empresas privadas especializadas, mediante a assistência *homecare*. O perfil de óbitos domiciliares identificado no período pré-pandêmico - maior frequência de óbitos de pessoas idosas, de raça/cor branca, com maior escolaridade e aumento das causas de óbito relacionadas ao aparelho circulatório e doenças endócrinas - é um padrão compatível com o relatado na literatura, de aumento da mortalidade domiciliar como uma opção, principalmente para pessoas com doenças crônicas em fase terminal em cuidados paliativos ^21^. Estudos brasileiros sobre o tema, realizados antes da pandemia da COVID-19, também mostraram maior frequência de óbitos domiciliares nos grupos de classe média e classe média alta ^22^, com maior ocorrência de óbitos hospitalares em pessoas mais pobres e vulneráveis ^23^.

Já o *cluster* secundário engloba RAs com baixo IPS. A mudança observada no perfil de casos e óbitos domiciliares no primeiro ano pandêmico, com aumento em pessoas mais jovens, principalmente nas faixas etárias produtivas; em homens; em pessoas de menor escolaridade; e de raça/cor da pele preta sugere que o óbito domiciliar durante o primeiro ano da pandemia pode ser reflexo de desassistência, afetando residentes de regiões de maior vulnerabilidade social ^10^ ^,^ ^24^. Maior mortalidade por COVID-19 em municípios com maior vulnerabilidade social e econômica foi relatado por Baggio et al. ^25^ em estudo que analisou os preditores de mortalidade dos casos de COVID-19 no Estado de Alagoas durante o processo de interiorização da pandemia. Nos Estados Unidos, Stokes et al. ^26^ verificaram que o excesso de óbitos não atribuíveis ao COVID-19, em 2020, foi significativamente mais elevado em municípios com menor renda familiar, menor nível de escolaridade e com sistema de saúde mais precário. Especificamente para óbitos domiciliares, resultado contrário ao deste estudo foi observado na Inglaterra, País de Gales, Escócia e Irlanda do Norte no primeiro ano da pandemia, no qual foi observado aumento da mortalidade domiciliar em locais com menor privação, exacerbando iniquidades no local de ocorrência do óbito já existentes antes da pandemia ^16^.

Este estudo tem algumas limitações. Foi utilizada a RA como unidade de análise espacial, evitando os pequenos números de óbitos, caso fosse utilizada a análise por unidades territoriais menores. Entretanto, as RAs do Município do Rio de Janeiro englobam bairros heterogêneos quanto às características socioeconômicas. Dessa forma, uma RA com IPS elevado pode conter territórios com maior vulnerabilidade social. Foi utilizada a categorização da escolaridade disponível nas declarações de óbito, sendo o ponto de corte de “12 anos de estudo ou mais” inadequado para indivíduos com menos de 18 anos, já que estes não teriam como alcançar essa escolaridade. Portanto, a análise da proporção de óbitos domiciliares segundo escolaridade pode ser afetada pela distribuição dos óbitos segundo faixa etária. Ressalta-se, entretanto, que houve redução da TMD em indivíduos de 15-19 anos e redução da proporção de óbitos naqueles com 4-7 anos de estudo, mas aumento da proporção de óbitos em indivíduos com 0-3 anos de estudo, sugerindo que a maior ocorrência de óbitos em pessoas com menos anos de estudo não é decorrente apenas de mudanças na distribuição de óbitos segundo faixa etária. O óbito domiciliar foi utilizado como *proxy* de óbitos sem assistência. Entretanto, o SIM não tem um campo que identifique se o óbito foi assistido por equipe de saúde, seja esta pública ou particular. Sendo assim, óbitos domiciliares podem ocorrer com assistência médica, e a falta desta informação dificultou a interpretação do aumento da taxa de mortalidade identificada no *cluster* primário no período 2016-2019, e se ela poderia ser um reflexo de melhoria na qualidade de fim de vida com assistência domiciliar em saúde. Por outro lado, optamos por não analisar os óbitos ocorridos em via pública, já que eles incluem óbitos ocorridos em ambulâncias, o que não refletiria falta de assistência. Entretanto, no ano 2020, o maior aumento proporcional da taxa de mortalidade foi observado em óbitos em via pública, e é possível que parte desses óbitos tenha ocorrido sem assistência. Informações populacionais segundo raça/cor e escolaridade não estavam disponíveis para os anos analisados, o que impossibilitou o cálculo das TMDs específicas. Entretanto, a análise da distribuição proporcional segundo raça/cor e escolaridade permitiu identificar variação na proporção observada no período pré-pandêmico e no primeiro ano da pandemia. Por fim, por tratar-se de um estudo ecológico, resultados observados no nível agregado não podem ser extrapolados para o nível individual. Portanto, a hipótese aventada de aumento da mortalidade domiciliar em populações com maior vulnerabilidade social e menor acesso a serviços de saúde deve ser confirmada em estudos futuros.

## Conclusão

Os resultados deste estudo demostram um excesso de mortalidade em hospitais, outros estabelecimentos de saúde, domicílio e via pública no Município do Rio de Janeiro em 2020, no primeiro ano da pandemia da COVID-19. Observou-se mudança no perfil de casos e das causas do óbito domiciliar, bem como aumento da TMD em RAs com menor IPS, sugerindo aumento da mortalidade domiciliar em populações mais vulneráveis socialmente e com menor acesso a serviços de saúde, hipótese que deve ser confirmada em estudos futuros.
